# Health technology assessment-informed pricing negotiation in China: higher negotiated price for more effective targeted anticancer medicines?

**DOI:** 10.1186/s12961-021-00810-1

**Published:** 2022-01-03

**Authors:** Cong Huang, Carolina Oi Lam Ung, Haishaerjiang Wushouer, Lin Bai, Tao Huang, Xinyi Li, Xiaodong Guan, Luwen Shi

**Affiliations:** 1 Department of Pharmacy Administration and Clinical Pharmacy, School of Pharmaceutical Sciences, Peking University, Haidian District, Xueyuan Road No. 38, Beijing, 100191 China; 2 State Key Laboratory of Quality Research in Chinese Medicine, Institute of Chinese Medical Sciences, University of Macau, Macao, 999078 China; 3 International Research Center for Medicinal Administration, Peking University, Beijing, China; 4 Center for Strategic Studies, Chinese Academy of Engineering, No. 2 Bingjiaokou HuTong, Xicheng District, Beijing, 100088 China

**Keywords:** Health technology assessment, Targeted anticancer medicines, Pricing negotiation

## Abstract

**Background:**

In China, health technology assessment (HTA) has recently been adopted in pricing negotiation for medicine listing in the National Reimbursement Drug List. At present, how HTA is applied to inform the decision-making process remains underreported. In order to explore how the adoption of HTA was translated into listing and price negotiation results in light of the confidential nature of the negotiating process, this study aimed to compare the negotiated price and the clinical benefit of selected targeted anticancer medicines (TAMs) involved in the 2019 negotiation.

**Main text:**

Among 16 TAMs successfully negotiated, only four TAMs representing four indication groups had appropriate reference medicines for comparison and were, therefore, included in the analysis. The price and clinical benefit of the four TAMs were compared against one or two reference medicines with the same initial indications. The sales prices for nine TAMs before and after the negotiation were extracted from the centralized medication procurement system. Clinical benefits were evaluated based on evidence from published articles and clinical guidelines. The results suggested that, despite the application of HTA, both rational and irrational decisions had been made about the reimbursement of TAMs in the 2019 negotiation, warranting further investigation.

**Conclusion:**

While the development and adoption of HTA has seen significant progress in China, actions are needed to ensure that the adoption of HTA is effectively applied in decisions on the reimbursement of medicines.

**Supplementary Information:**

The online version contains supplementary material available at 10.1186/s12961-021-00810-1.

## Background

Health technology assessment (HTA) is a multidisciplinary process that uses explicit methods to determine the value of a health technology at different points in its life cycle [1]. The purpose of HTA is to inform decision-making in order to promote an equitable, efficient and high-quality health system. It has become a standard policy tool for informing decision-makers about managing the listing, reimbursement and recommended use of pharmaceuticals, medical devices and other technologies within health systems [2]. Many countries, including the United States, Germany, Australia and New Zealand, have employed HTA to inform the healthcare decision-making process as an effort to control rapidly increasing healthcare costs [3]. However, the progress from development through adoption to translation of HTA has been historically slow in Asia [4].

In China, HTA was introduced as early as the 1980s. The purpose was to enhance medicine accessibility and affordability to support the primary goals of health reform in achieving universal health coverage and health for all in the country [5]. Despite the significant progress made in the overall development in terms of capacity-building and research, whether HTA has been fully integrated into the policy cycle to inform drug policies and regulations remains questionable, and the impact of HTA on drug listing and price negotiation is underreported [6].

The National Reimbursement Drug List (NRDL) was formally established in 2000 by the Ministry of Human Resources and Social Security (MHRSS) in China. The decisions about drug inclusion and pricing on the NRDL were made based on a comprehensive criteria framework which incorporated clinical needs, safety, efficacy, pricing, cost and cost-effectiveness [7]. Despite the emphasis on cost-effectiveness in the evaluation, it was not until the third revision of the NRDL in 2017 that pharmacoeconomic evaluation was adopted for the first time by the MHRSS as a negotiation tool during the decision-making process for medicine listing and pricing negotiation [8].

On 28 November 2019, the National Healthcare Security Administration (NHSA) released the “Notice on Including Year 2019 Negotiated Medicines in ‘National Basic Medical Insurance’”. This marked a crucial step in the deepening of HTA adoption in China drug policy, as it introduced a new centralized strategic price negotiation process which employed the parallel calculation of the ceiling price and the method of competitive negotiations. The policy placed more emphasis on evidence-based HTA data than the face-to-face bargaining process during the decision-making process of medicine inclusion and price negotiation [8, 9]. According to the results from the fourth national drug pricing negotiation, the prices of new negotiated medicines were generally reduced by 60.7% on average [10].

While the overall outcomes of the fourth pricing negotiation appear to favour accessibility and affordability of the newer innovative medicines, the negotiation process was kept entirely confidential, and how HTA was applied and translated into the final decisions remains unknown. Among the negotiated medicines, targeted anticancer medicines (TAMs) received much attention due to “life-saving” value and skyrocketing prices [11]. In this study, we intended to explore how the adoption of HTA was translated into enlistment and price negotiation results by comparing the negotiated prices and the clinical benefits of selected TAMs involved in the 2019 negotiation. The analysis of HTA adoption and translation specific to a drug group will shed light on how to better ensure the HTA evidence base for decision-making related to health technology in the future for China and other recent HTA adopters alike.

## Methods

During the fourth pricing negotiation, upon reaching a consensus on the drug price by the marketing authorization holders and the government, 16 TAMs were successfully negotiated and thus included in the NRDL. Among them, only four TAMs representing four indication groups had appropriate reference medicines for comparison and were, therefore, included in the analysis. These included sintilimab (for relapsed Hodgkin lymphoma), alectinib (for anaplastic lymphoma kinase [ALK]-positive metastatic non-small cell lung cancer [NSCLC]), pyrotinib (for human epidermal growth factor receptor-2 [HER2]-positive relapsed or metastatic breast cancer) and erlotinib (for epidermal growth factor receptor [EGFR] mutation-positive advanced NSCLC). Each of the four TAMs was compared against one or two reference medicines which were approved by the National Medical Products Administration (NMPA) to be used for the same initial cancer indications [12]. The reference medicines were camrelizumab (vs sintilimab), crizotinib (vs alectinib), lapatinib (vs pyrotinib) and icotinib and gefitinib (vs erlotinib). The information for all 16 successfully negotiated TAMs is presented in Additional file 1: Online Appendix 1.

The sales prices for the nine TAMs from the four comparison groups before and after the negotiation were extracted from the centralized procurement system for medicines which displayed the official sales prices set by the pricing authorities [13]. The daily cost of each TAM was calculated based on the prescription and dosage information indicated on the product insert approved by the NMPA.

Clinical benefits were evaluated based on a thorough review of the current evidence from meta-analysis or clinical trials conducted in Chinese or other Asian patient populations published up to 28 November 2019. The detailed search strategies were provided in Additional file 2: Online Appendix 2. As such, only the highest-level evidence available was used to compare the clinical benefits of TAMs from each group [14]. Recommendations from the latest clinical guidelines by the Chinese Society of Clinical Oncology (CSCO) and the US National Comprehensive Cancer Network (NCCN) in 2019 were also compared. All expense data was reported in US dollars using the exchange rate of US$ 1 = ¥6.8985 in 2019 [15]. Further information about the nine TAMS from the four indications groups is provided in Table 1.

**Table 1:** Basic information and clinical benefit evidence for four groups of nine targeted anticancer medicines

Group	Generic name	Marketing authorization holder	Therapeutic class^a^	ATC code	CSCO guideline^b^ recommendation	NCCN guideline^c^ recommendation	Clinical benefit comparison	Category of evidence
1	Sintilimab	Innovent	Hodgkin lymphoma	/	Recommended	Not mentioned	Sintilimab: OR 80.4%; CR 34% Camrelizumab: OR 76.0%; CR 28.0%	Two single-arm clinical trials [16, 17]
	Camrelizumab (reference)	HengRui		/	Not mentioned	Not mentioned		
2	Alectinib	Roche	NSCLC	L01XE36	First-line therapy Preferred	First-line therapy Preferred	Alectinib vs crizotinib PFS (months): NE vs 10.2, *p* < 0.001	Randomized controlled trial [18]
	Crizotinib (reference)	Pfizer		L01XE16	First-line therapy Recommended	First-line therapy Other Recommended		
3	Pyrotinib	HengRui	Breast cancer	/	Second-line therapy Recommended	Not Mentioned	Pyrotinib vs lapatinib ORR: 78.5% vs 57.1, *p* = 0.01 PFS (months): 18.1 vs 7.0, *p* < 0.001	Randomized controlled trial [19]
	Lapatinib (reference)	GlaxoSmithKline		L01XE07	Second-line therapy Preferred	Other Recommended		
4	Erlotinib	Roche	NSCLC	L01XE03	First-line therapy Recommended	First-line therapy Other Recommended	No significant difference in PFS and overall survival	Network meta-analysis [20]
	Icotinib (reference)	Betta		/	First-line therapy Recommended	Not Mentioned		
	Gefitinib (reference)	AstraZeneca		L01XE02	First-line therapy Recommended	First-line therapy Other Recommended		

*NSCLC* non-small cell lung cancer, *ATC* Anatomical Therapeutic Chemical classification, *OR* objective response, *CR* complete remission, *PFS* progression-free survival, *NE* not estimable, *ORR* overall response rate

^a^Therapeutic class: summarized from indications in the manufacturers’ instructions of products approved by NMPA

^b^CSCO guideline: 2019 Chinese Society of Clinical Oncology clinical guidelines in oncology [21–23]

^c^NCCN guideline: 2019 National Comprehensive Cancer Network clinical guidelines in oncology [24, 25]

## Findings

In this study, high-priced TAMs were selected to explore the possible impact of HTA adopted in the pricing negotiation process on the treatment daily costs. Four sets of TAMs were compared based on their clinical benefits, and four different scenarios of HTA adoption and translation were identified: (1) the TAM with more favourable clinical benefit was listed on the NRDL and resulted in lower daily cost, whereas the reference TAM with less favourable clinical benefit failed the pricing negotiation; (2) the TAMs with different clinical benefits were both listed on the NRDL, but the one with more favourable clinical benefits was negotiated at a higher price, resulting in a higher daily treatment cost; (3) despite conflicting indications of clinical benefits, one TAM was listed on the NRDL at a negotiated price that resulted in a higher daily treatment cost compared to the comparator TAM not listed on the NRDL; and (4) the TAMs with similar clinical benefits were all listed on the NRDL, but each was negotiated at different prices, resulting in wide variations in daily treatment costs.

### Scenario 1: sintilimab vs camrelizumab

**Fig. 1:**
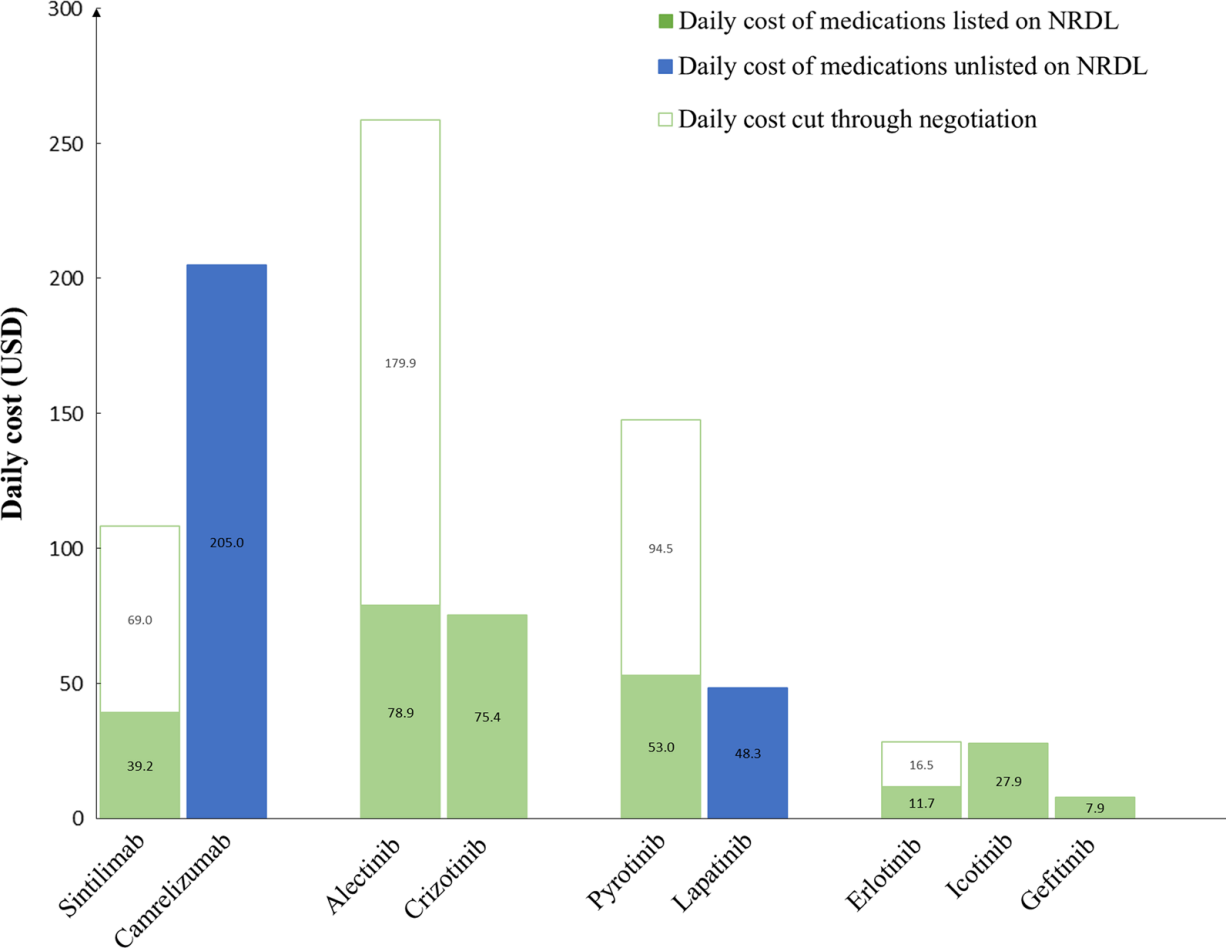
Daily cost of four groups of nine targeted anticancer medicines. *NRDL* National Reimbursement Drug List

Sintilimab and camrelizumab were both approved for patients with relapsed or refractory classical Hodgkin lymphoma after two or more lines of therapy. A multicentre, single-arm, phase 2 trial showed that sintilimab was effective among Chinese patients, with 74 (80%) of 92 patients showing an objective response (OR) and 31 (34%) demonstrated complete remission (CR) [16, 17]. As compared to the results from another multicentre, single-arm, phase 2 trial, camrelizumab demonstrated a lower response rate in Chinese patients, in which only 57 (76%) of 75 patients achieved an OR, including 21 (28%) patients who achieved CR [16, 17]. Based on these results, sintilimab was recommended over camrelizumab by the CSCO guideline due to the superior clinical benefit. After negotiation, only sintilimab with better clinical benefit was successfully negotiated with a price cut of more than 64%, and the daily cost of sintilimab is now about one fifth that of camrelizumab (Fig. 1).

### Scenario 2: alectinib vs crizotinib

Alectinib, a potent, highly selective central nervous system (CNS)-active inhibitor of ALK, was compared with crizotinib, the first ALK inhibitor and the standard of care, for their clinical benefits for patients with ALK-positive metastatic NSCLC. Both medicines were recommended by the CSCO guideline, but alectinib was the medicine of choice according to the NCCN guideline. In an open-label, randomized phase 3 trial, alectinib and crizotinib underwent a head-to-head comparison. The study showed that alectinib had more favourable progression-free survival (PFS) that was consistent across different patient subgroups [18]. After negotiation, alectinib was successfully negotiated, with a price cut of almost 70%, and the daily cost was just US$ 4.5 higher than crizotinib, which remained listed on the NRDL (Fig. 1).

### Scenario 3: pyrotinib vs lapatinib

Pyrotinib, an irreversible pan-ErbB inhibitor, and lapatinib, an HER2 tyrosine kinase inhibitor (TKI), were both approved for patients with HER2-positive relapsed or metastatic breast cancer. In a randomized, phase II study, pyrotinib plus capecitabine yielded a statistically significant better overall response rate (78.5% vs 57.1%) and PFS (18.1 vs 7.0 months) than lapatinib plus capecitabine in women with HER2-positive metastatic breast cancer previously treated with taxanes, anthracyclines and/or trastuzumab [19]. Although pyrotinib was recommended as a second-line treatment and lapatinib was recommended as a preferred second-line treatment in the CSCO guideline, emerging clinical data suggested that pyrotinib was more superior than lapatinib in terms of clinical benefits. Nevertheless, pyrotinib was successfully negotiated with higher daily cost than lapatinib which was no longer listed on the NRDL (Fig. 1).

### Scenario 4: erlotinib vs icotinib, gefitinib

Erlotinib, icotinib and gefitinib, known as the first-generation EGFR TKIs, are target regimens for patients with advanced EGFR-mutated NSCLC. All three medicines were recommended as first-line treatment in the CSCO guideline, whereas only erlotinib and gefitinib were considered first-line treatment in the NCCN guideline. As shown in a network meta-analysis study, all three TKIs showed favourable and comparable PFS and overall survival relative to EGFR-TKI monotherapy [20]. After negotiation, all three treatments were listed on the NRDL, with erlotinib being successfully renegotiated with a price cut of more than 58%, much lower than icotinib, yet still higher than gefitinib (Fig. 1).

## Discussion

HTA is a useful tool to evaluate the clinical and economic value of negotiated medicines, helping improve patients’ access to cost-effective medicines [26]. Despite the recent development of HTA in China, the findings of this study suggest that the translation of the HTA evidence base in the decisions made about the drug enlistment and price negotiation was not fully demonstrated. In-depth analysis of four selected TAMs showed a lack of consistency and a deficit of HTA performance in rationalizing the enlistment decisions in the fourth pricing negotiation.

While recommended treatments such as sintilimab and alectinib were successfully negotiated and patients had improved access to their needed medicines with lower out-of-pocket expenses [27], lapatinib, a recommended treatment associated with a lower daily cost, was excluded from the NRDL. The exclusion of lapatinib from the NRDL was particularly concerning for both the clinicians and the patients. This medicine is recommended by the CSCO guideline as the preferred second-line therapy for HER2-positive relapsed or metastatic breast cancer, whereas the newly listed medicine indicated for the same conditions, pyrotinib, is not. These observations reconfirmed the previous findings that HTA has not been fully and effectively employed in the reimbursement and pricing negotiation process by the authorities.

Mismatch between clinical evidence and decisions made about drug enlistment and price negotiation can easily cause confusion among clinicians and patients. For health technology-related decision-making to be truly evidence-based, a clearly defined framework is needed for formally institutionalized HTA allowing effective exchange, synthesis and ethically sound application of evidence and scientific knowledge throughout the decision-making process by the policy-makers [28]. In many high-income countries, the efficacy, safety and value of medical innovations are assessed through a formal HTA system [29]. Government initiatives that institutionalize HTA, such as establishing a national HTA system or consortium, should be considered to reinforce not only the development, but also, more importantly, the adoption and translation of HTA in China [6]. Moreover, no valid standards have been developed to evaluate the clinical benefits of cancer therapies in China. Official frameworks with algorithmic scales are needed for the clinical benefit assessment of cancer therapies and other health economic evaluations, like the American Society of Clinical Oncology Value Framework (ASCO-VF) or the European Society for Medical Oncology Magnitude of Clinical Benefit Scale (ESMO-MCBS) [30].

In addition, icotinib, with clinical benefits only comparable to its reference medicine, was successfully negotiated at a much higher price. Considering icotinib, gefitinib and erlotinib are of comparable clinical benefits, the decision-making of pricing negotiation resulting in various levels of price cut appeared to deviate from the value-based price standard [31]. Collectively, this may be explained at least partly by the lack of other price regulation mechanisms for medicines after successful listing in the NRDL in 2016, leading to a stagnation of price adjustment despite emerging clinical evidence on the available treatments. To resolve this deficiency, HTA should be adopted fully and dynamically for the re-evaluation of the clinical and economic value of medicines already listed. Moreover, with the Chinese health insurance payment reform in progress, centralized regulation of reimbursement and pricing should be recommended and implemented with reference price clusters in a way similar to Germany in order to decrease unnecessary expenditures of the health insurance funding [32, 33].

There were several limitations in this study. First, the price information was collected from the centralized procurement system due to the nondisclosure agreement with enterprises during price negotiation. However, it is highly possible that the procured price was equal to the negotiated price in China’s setting, and that the publicly negotiated prices of two TAMs were truly consistent with the prices listed in the centralized procurement system. Second, in the absence of a standard framework for evaluation of cancer therapies in China, we evaluated clinical benefits roughly based on the evidence about their efficacy from published articles and clinical guidelines. Third, the negotiation process was kept entirely confidential, thus we had no access to any information about the actual translating-to-decision process. The findings of the current study can only indicate possible concerns about the HTA application, and cannot fully assess the effectiveness of HTA adoption and the influencing factors. In addition, using the clinical recommendations of NCCN guidelines as a reference has prevented the comparison of medicines which were developed in China but not subject to Food and Drug Administration (FDA) approval, and might introduce some bias. Third, as the medicines included in this study were limited to TAMs, further evidence in other therapeutic classes would be needed to verify the findings and the implications. However, experiences in oncology were likely to reflect the wider issues surrounding the introduction of new, innovative and expensive therapies [29].

## Conclusion

The principles underlying the translation of HTA included decision-making that was well informed by sound scientific evidence provided by experts, and made in a consistent manner with high transparency and fairness. For HTA to be optimally adopted to facilitate the utilization of better medicines at rational prices to address evolving unmet medical needs, initiatives to strengthen the process, procedure and guidance of HTA translation at different levels of authority are important. Specific measures such as a framework for benefit evaluation and reference price clusters should be the priority.

## Supplementary Information

**Additional file 1: Appendix 1**: The information of all 16 targeted anticancer medicines successfully negotiated.

**Additional file 2: Appendix 2**: Search strategies.

## Data Availability

The datasets used and/or analysed during the current study are available from the corresponding author on reasonable request.

## Abbreviations

ALK: Anaplastic lymphoma kinase
ASCO-VF: American Society of Clinical Oncology Value Framework
CR: Complete remission
CSCO: Chinese Society of Clinical Oncology
EGFR: Epidermal growth factor receptor
ESMO-MCBS: European Society for Medical Oncology Magnitude of Clinical Benefit Scale
HER2: Human epidermal growth factor receptor-2
HTA: Health technology assessment
MHRSS: Ministry of Human Resources and Social Security
NCCN: National Comprehensive Cancer Network
NHSA: National Healthcare Security Administration
NMPA: National Medical Products Administration
NRDL: National Reimbursement Drug List
NSCLC: Non-small cell lung cancer
OR: Objective response
ORR: Overall response rate
PFS: Progression-free survival
TAMs: Targeted anticancer medicines
