# Supplementary material for: Health technology assessment-informed pricing negotiation in China: higher negotiated price for more effective targeted anticancer medicines?
Source: Health Res Policy Syst. 2022 Jan 3;20:3. doi: 10.1186/s12961-021-00810-1 (PMC8722031; doi:10.1186/s12961-021-00810-1)
Supplement: Supplementary file 1 — Additional file 1: Appendix 1: The information of all 16 targeted anticancer medicines successfully negotiated. [file 12961_2021_810_MOESM1_ESM.docx]

## Appendix 1: The information of all 16 targeted anticancer medicines successfully negotiated

| **Selected in study** | **No.** | **Generic name** | **Cancer site** | **Indications** | **Reference medicine** | **Reasons for choosing the reference medicines or not including the medicines** |
| --- | --- | --- | --- | --- | --- | --- |
| Scenario 1 | 1 | sintilimab | Lymphoma | Classical Hodgkin lymphoma that was relapsed or refractory after two or more lines of therapy | camrelizumab | the same Indication |
| **Scenario 2** | 2 | alectinib | Lung | A kinase inhibitor indicated for the treatment of patients with anaplastic lymphoma kinase (ALK)-positive metastatic non-small cell lung cancer (NSCLC) | crizotinib | the same Indication |
|  |  |  |  |  |  | **Ceritinib (not included)** with the same indication and (ALK)-positive metastatic non-small cell lung cancer (NSCLC) who have progressed on or are intolerant to crizotinib. |
| **Scenario 3** | 3 | pyrotinib | Breast | A kinase inhibitor indicated in combination with: capecitabine, for the treatment of patients with advanced or metastatic breast cancer whose tumors overexpress human epidermal growth factor receptor 2(HER2) and who have received prior therapy including an anthracycline, a taxane, and trastuzumab. | lapatinib | the same Indication |
| **Scenario 4** | 4 | erlotinib | Lung | A kinase inhibitor indicated for: first-line treatment of patients with metastatic non-small cell lung cancer (NSCLC) whose tumors have epidermal growth factor receptor (EGFR) mutations. | gefitinib  icotinib | the same Indication |
| NA | 5 | fruquintinib | Colorectal | A kinase inhibitor indicated for the treatment of patients with: Metastatic colorectal cancer (CRC) who have been previously treated with fluoropyrimidine-, oxaliplatin- and irinotecan-based chemotherapy, an antiVEGF therapy, and, if RAS wild-type, an anti-EGFR therapy. | NA | .  No medicines on the market with the same indications |
| NA | 6 | apatinib | Gastric | Metastatic or relapsed gastric cancer or adenocarcinoma of esophagogastric junction after 5 or more lines of therapy. | NA |  |
| NA | 7 | bevacizumab | Colorectal Lung | Metastatic colorectal cancer, in combination with intravenous 5-fluorouracil-based chemotherapy for first- or second-line treatment.  Unresectable, locally advanced, recurrent or metastatic non-squamous non-small cell lung cancer, in combination with carboplatin and paclitaxel for first-line treatment. | NA |  |
| NA | 8 | chidamide | Lymphoma | Peripheral T-cell lymphomas that was relapsed or refractory after one or more lines of intravenous chemotherapy. | NA |  |
| NA | 9 | everolimus | Kidney Pancreatic Brain | Adults with advanced renal cell carcinoma (RCC) after failure of treatment with sunitinib or sorafenib.  Adults with progressive neuroendocrine tumors of pancreatic origin (PNET) and adults with progressive, well-differentiated, non-functional neuroendocrine tumors (NET) of gastrointestinal (GI) or lung origin that are unresectable, locally advanced or metastatic.  A Kinase inhibitor indicated for the treatment of adult and pediatric patients aged 1 year and older with TSC who have subependymal giant cell astrocytoma (SEGA) that requires therapeutic intervention but cannot be curatively resected.  A kinase inhibitor indicated for the adjunctive treatment of adult and pediatric patients aged 2 years and older with TSCassociated partial-onset seizures. | NA |  |
| NA | 10 | nimotuzumab | Head and neck | In combination with radiotherapy for treatment of patients with stage III/IV nasopharyngeal carcinoma whose tumors have epidermal growth factor receptor (EGFR) mutations. | NA |  |
| NA | 11 | olaparib | Ovarian epithelial  fallopian tube  primary peritoneal | For the maintenance treatment of adult patients with recurrent epithelial ovarian, fallopian tube or primary peritoneal cancer, who are in a complete or partial response to platinum-based chemotherapy | NA |  |
| NA | 12 | pertuzumab | Breast | Use in combination with trastuzumab and chemotherapy as: neoadjuvant treatment of patients with HER2-positive, locally advanced, inflammatory, or early stage breast cancer (either greater than 2 cm in diameter or node positive) as part of a complete treatment regimen for early breast cancer; adjuvant treatment of patients with HER2-positive early breast cancer at high risk of recurrence. | NA |  |
| NA | 13 | Recombinant Human Endostatin | Lung | In combination with NP chemotherapy for initial treatment or retreatment of patients with stage III/IV non-small cell lung cancer (NSCLC) | NA |  |
| NA | 14 | ruxolitinib | Myelodysplastic  myeloproliferative disorders | Intermediate or high-risk myelofibrosis, including primary myelofibrosis, post-polycythemia vera myelofibrosis and post-essential thrombocythemia myelofibrosis | NA |  |
| NA | 15 | sorafenib | Kidney Liver Thyroid | Unresectable hepatocellular carcinoma; Advanced renal cell carcinoma; Locally recurrent or metastatic, progressive, differentiated thyroid carcinoma refractory to radioactive iodine treatment | NA |  |
| NA | 16 | trastuzumab | Breast Gastric | The treatment of HER2-overexpressing breast cancer.  The treatment of HER2-overexpressing metastatic gastric or gastroesophageal junction adenocarcinoma | NA |  |
