# Supplementary material for: Health technology assessment-informed pricing negotiation in China: higher negotiated price for more effective targeted anticancer medicines?
Source: Health Res Policy Syst. 2022 Jan 3;20:3. doi: 10.1186/s12961-021-00810-1 (PMC8722031; doi:10.1186/s12961-021-00810-1)
Supplement: Supplementary file 2 — Additional file 2: Appendix 2: Search strategies. [file 12961_2021_810_MOESM2_ESM.docx]

## Appendix 2: Search strategies

| Search date | Database | Search strategies |
| --- | --- | --- |
| 1/31/2020 | Pubmed | ((sintilimab[Title/Abstract] OR camrelizumab[Title/Abstract] OR alectinib[Title/Abstract] OR crizotinib[Title/Abstract] OR pyrotinib[Title/Abstract] OR lapatinib[Title/Abstract] OR erlotinib[Title/Abstract] OR icotinib[Title/Abstract] OR gefitinib[Title/Abstract]) AND (efficacy[Title/Abstract] OR effectiveness[Title/Abstract] OR benefit* [Title/Abstract])) AND (clinical trial*[Title/Abstract] OR randomized controlled trial*[Title/Abstract] OR RCT*[Title/Abstract] OR phase[Title/Abstract] OR meta analysis[Title/Abstract] OR meta-analysis[Title/Abstract]) |
| 1/31/2020 | Embase | #1 sintilimab:ab,ti OR camrelizumab:ab,ti OR alectinib:ab,ti OR crizotinib:ab,ti OR pyrotinib:ab,ti OR lapatinib:ab,ti OR erlotinib:ab,ti OR icotinib:ab,ti OR gefitinib:ab,ti  #2 efficacy:ab,ti OR effectiveness:ab,ti OR benefit*:ab,ti  #3 'clinical trial*':ab,ti OR 'randomized controlled trial*':ab,ti OR RCT*:ab,ti OR phase:ab,ti OR 'meta analysis':ab,ti OR 'meta-analysis':ab,ti  #4 #1 AND #2 AND #3 |
| 1/31/2020 | Cochrane Library | #1 (sintilimab OR camrelizumab OR alectinib OR crizotinib OR pyrotinib OR lapatinib OR erlotinib OR icotinib OR gefitinib):ab,ti  #2 (efficacy OR effectiveness OR benefit*):ab,ti  #3 (clinical trial* OR randomized controlled trial* OR RCT* OR phase OR meta analysis OR meta-analysis):ab,ti  #4 #1 AND #2 AND #3 |
| 1/31/2020 | Web of Science | AB=( sintilimab OR camrelizumab OR alectinib OR crizotinib OR pyrotinib OR lapatinib OR erlotinib OR icotinib OR gefitinib) AND AB=( efficacy OR effectiveness OR benefit*) AND AB=( "clinical trial*" OR "randomized controlled trial*" OR RCT* OR phase OR "meta analysis" OR "meta-analysis")  Choose English |
